# Long term follow-up demonstrating stability and patient satisfaction of minimally invasive punch technique for percutaneous bone anchored hearing devices

**DOI:** 10.1186/s40463-018-0316-5

**Published:** 2018-11-20

**Authors:** Yaeesh Sardiwalla, Nicholas Jufas, David P. Morris

**Affiliations:** 1 Faculty of Medicine, Dalhousie University, Halifax, NS Canada; 2 Division of Otolaryngology – Head and Neck Surgery, Dalhousie University, Halifax, NS Canada; 3 Discipline of Surgery, Sydney Medical School, University of Sydney, Sydney, Australia; 4 Department of Otolaryngology – Head and Neck Surgery, Faculty of Medicine and Health Sciences, Macquarie University, Sydney, Australia; 5 QEII Health Science Center - VG Site Otolaryngology, 5820 University Ave - Rm 3037, Halifax, NS B3H 2Y9 Canada

**Keywords:** Patient safety, Otologic surgical procedures, Bone conduction, Minimally invasive surgical procedures, Patient satisfaction, Quality improvement

## Abstract

**Objective:**

Minimally Invasive Ponto Surgery (MIPS) was recently described to facilitate the placement of percutaneous bone anchored hearing devices. As early adopters of this new procedure, we sought to perform a quality assurance project using our own small prospective cohort to justify this change in practice. We chose to examine device stability and to gauge our patients’ perspective of the surgery and their overall satisfaction with the process.

**Methods:**

A total of 12 adult patients who underwent MIPS between 2016 and 2017 with a minimum post-operative follow-up of 12 months were included in this study. A prospective MIPS research clinic was used to follow patients, assess the implant site soft tissue status and gather qualitative information through patient interviews and surveys.

**Results:**

The mean (SD) soft tissue status score averages using the IPS Scale were low for inflammation 0.1 (0.1), pain 0.1 (0.1), skin height 0.2 (0.1) and total IPS score 0.4 (0.3) indicating minimal soft tissue changes. Patient experiences with MIPS were overwhelmingly positive in reports through the MIPS modified SSQ-8. All patients reported speedy recoveries and no long-term complications. There were zero device losses.

**Conclusion:**

The series presented in this paper represents the first MIPS cohort with long term follow-up to be published to date in North America. Our findings conclude both device stability and patient satisfaction with no loss of fixtures. Consequently, we have adopted MIPS as our procedure of choice for the placement of all percutaneous BAHDs.

## Introduction

In 2011, Hultcrantz et al. described the Minimally Invasive Ponto Surgery (MIPS) procedure where a 5 mm dermal punch was used to remove the limited tract of soft tissue needed to accommodate the Ponto (Oticon, Copenhagen, Denmark) abutment [1]. The drilling procedure could then be completed in seconds through a cannula placed to protect the skin and soft tissues while holding cooling fluid. MIPS heralded a departure from the traditional “open approach” to percutaneous fixture placement. Soft tissue preservation and longer abutments placed in a few simple surgical steps marked a significant evolution of the original surgical procedure. While keen to adopt this simplified approach, we were equally vigilant to discover whether such changes were possible without compromise to patient care.

In our groups previously published direct cost analysis, we calculated that MIPS offered a saving of approximately $450 per operation when compared to open approaches [2]. The majority of this saving stems from the large time saving with MIPS procedure (0.10 h) versus the open approach (1.13 h) [2]. We were also able to move bone anchored hearing device (BAHD) surgery out of the main operating room (OR) with this transition, making valuable OR time accessible for other services [2].

In this follow-up study, we sought to investigate the long-term stability of the fixture/abutment in our single-center Halifax cohort. Early evidence from soft tissue preserving techniques for BAHD have already suggested favorable outcomes [3, 4]. To date, there have been few MIPS case series published. Bonilla et al. showed promising MIPS outcomes in their cohort, confirming a shorter procedure time and fewer skin complications one week after surgery when compared to the linear incision technique [5]. While details of other experiences are limited, there are mixed results reported [6]. The highly anticipated long-term, multi-centre MIPS outcomes data recently published showed no difference in inflammation compared to linear incisions [7–9]. There was improvement in skin sensation, sagging, cosmetic result and reduction of surgical time but a non-significant increase in implant extrusion rate that warrants further investigation [7–9].

Generally, it is accepted that osseointegration and implant stability occurs within 3 months for adults and 6 months for children [10–12]. This period has traditionally been used to guide the timing for loading the abutment. We reasoned that if the fixtures were to fail or have complications, they would do so early after surgery. We proposed a minimum follow-up period of 12 months to capture such failures.

As early adopters of this new procedure, we sought to perform a quality assurance project using our own small prospective cohort to justify this change in practice. As medical professionals, we balance the introduction of new techniques and intervention with patient safety, especially where large bodies of evidence do not yet exist [10]. We chose to examine device stability and to gauge our patients’ perspective of the surgery and their overall satisfaction with the process.

## Methods

### Patient selection and surgery

Institutional permission from Nova Scotia Health Authority Research Ethics Board was obtained to conduct the quality assurance of the new MIPS technique. A total of 12 sequential adult patients who underwent MIPS between 2016 and 2017 with a minimum post-operative follow-up of 12 months were prospectively enrolled in this study at the time of proceeding with surgery. All cases were performed or directly assisted and supervised by the same experienced consultant otologist. Specific training covering the MIPS procedure and novel drilling technique in both an animal and synthetic temporal bone model had been undertaken prior to the first real-time surgery. Exclusive MIPS cases were performed under local anesthetic. General anesthesia was reserved for those undergoing concomitant middle ear or mastoid surgery. Cases requiring general anesthesia were performed in the main OR. Most local anesthesia cases were performed in a minor procedures room conforming to Infection Prevention and Control standards. For an overview of the surgical technique and equipment required for MIPS, please review our groups previous article on this topic [2]. Sound processors were loaded at median of 6 weeks, with a range from 4 to 6 weeks. Table 1 shows complete demographic information.

**Table 1:** The demographic information and operative characteristics from the cohort of MIPS patients followed for a minimum of 12 months

Age	Sen	Previous Diagnosis	Follow-up Duration	Surgery	Admission	Anesthesia	Site
74	F	Psoriasis and Left Mastoid cavity	30 months	MIPS	Day Case	Local	Main OR
58	M	Chronic Suppurative Otitis Media - Previous mastoidectomy	30 months	MIPS	Day Case	General	Main OR
57	F	Chronic Suppurative Otitis Media	28 months	MIPS + Mastoid obliteration	Admitted	General	Main OR
58	M	Chronic Suppurative Otitis Media	24 months	MIPS	Day Case	Local	Main OR
28	F	Chronic Suppurative Otitis Media	23 months	MIPS + Mastoid obliteration	Day Case	General	Main OR
55	M	Chronic Suppurative Otitis Media	20 months	MIPS + Mastoid obliteration	Admitted	General	Main OR
55	M	Chronic Suppurative Otitis Media	18 months	MIPS and Left Tympanoplasty	Admitted	General	Main OR
52	F	Chronic Suppurative Otitis Media	14 months	MIPS + Blind Sac closure	Admitted	General	Main OR
30	M	Chronic Suppurative Otitis Media Tympanomastoidecomy	20 months	MIPS	Day Case	Local	Minor Procedures
65	F	CSOM preexisting mastoid cavity	20 months	MIPS	Day Case	Local	Minor Procedures
41	M	Right Microtia	19 months	MIPS	Day Case	Local	Minor Procedures
74	M	CSOM	17 months	MIPS	Day Case	Local	Minor Procedures

The details of each surgical case, fixture/abutment specifications, external BAHD chosen and operating time was recorded. Each patient’s surgical data is captured in Table 2. The MIPS operating time was calculated prospectively from the moment of skin punch, to the moment the healing cap was placed.

**Table 2:** Technical information relating to the MIPS surgery for each of the 12 patients. For each procedure 50 N.cm of torque was obtained. * Topical application of ciprofloxacin/dexamethasone drops (Ciprodex ®) around abutment

Sound Processor	Abutment (and change if applicable)	Implant	Length of Surgery (mins)	Skin thickness (mm)	Number of turns	Adjunctive Measures
Left Ponto Plus power	Ponto 9 mm -- > 12 mm	4 mm Ponto BHX	8.2	4.5	S	Abutment lengthened
Left BAHA S	Ponto 12 mm	4 mm Ponto BHX	9.S	7	4.5	Ciprodex* drops ‘local toilet’*
Left BAHA 5	Ponto 12 mm	4 mm Ponto BHX	4	6	S	Stay suture applied interiorly
Right Ponto Plus power	Ponto 9 mm	4 mm Wide Ponto	9.6	5	4.5	None
Left BAHA S	Ponto 12 mm -- > 9 mm	4 mm Wide Ponto	8	S	4.5	Abutment shortened Ciprodex* ‘local toilet’*
Right BAHA S	Ponto 12 mm	4 mm Wide Ponto	S.2	6	3.25	None
Right BAHA S	Ponto 12 mm	4 mm Ponto BHX	6	S	4.5	Direct trauma to implant, close examination - no apparent harm
Left BAHA S	Ponto 12 mm	4 mm Ponto BHX	4.2	6	5	Slight skin redundancy superiorly at implant site
Right Baha 5	Ponto 12 mm	4 mm Ponto BHX	7	7	4.5	None
Right Ponto Plus	Ponto 12 mm	4 mm Ponto BHX	8	6	4.25	None
Right Baha 5	Ponto 12 mm - > 9 mm	4 mm Ponto BHX	7	S	4.5	Abutment shortened
Right Ponto Plus	Ponto 9 mm	4 mm Ponto BHX	4.5	4	4.5	None

### Research clinic

Follow-up was prospective. A coordinated MIPS research clinic was used to follow patients, assess implant sites and gather qualitative information through patient interviews and surveys. Soft tissue status around the implant was evaluated independently by three different assessors (staff otologist, otology fellow and senior medical student) using the eight point Inflammation, Pain, Skin Height (IPS) Scale proposed by Kruyt [13]. The IPS scale was designed to assess long-term wound healing at the bone conduction site using objective and patient reported measures of inflammation (skin integrity, erythema, edema and granulation tissue), pain and skin height/numbness to prompt treatment decisions. This addresses the shortcomings of the Holgers scale such as dichotomous subjective responses, not considering long-term wound healing failures such as increased skin height and not encapsulating patient pain [13–15]. Patients qualitative perspectives were assessed using the Surgical Satisfaction Questionnaire (SSQ-8) modified for MIPS and through a semi-structured interview that assessed their experience. The modified SSQ-8 asked patients to use a Likert Scale (1 – very satisfied; 5 – very unsatisfied) to rate their MIPS surgical experience according 8 domains: the result of their surgery, their recovery, satisfaction with abutment length, appearance of screw, the follow-up they received, overall satisfaction and how likely they would be to recommend MIPS to others who require BAHD. Both the IPS scale and modified SSQ-8 were administered at the patient’s most recent follow-up visit.

### Statistical analysis

Data from all three observers’ IPS scores were analyzed independently and were averaged. Only descriptive analysis was used since this is a small sample evaluation. Box and whisker plots of survey data were used to present responses.

## Results

**Fig. 1:**
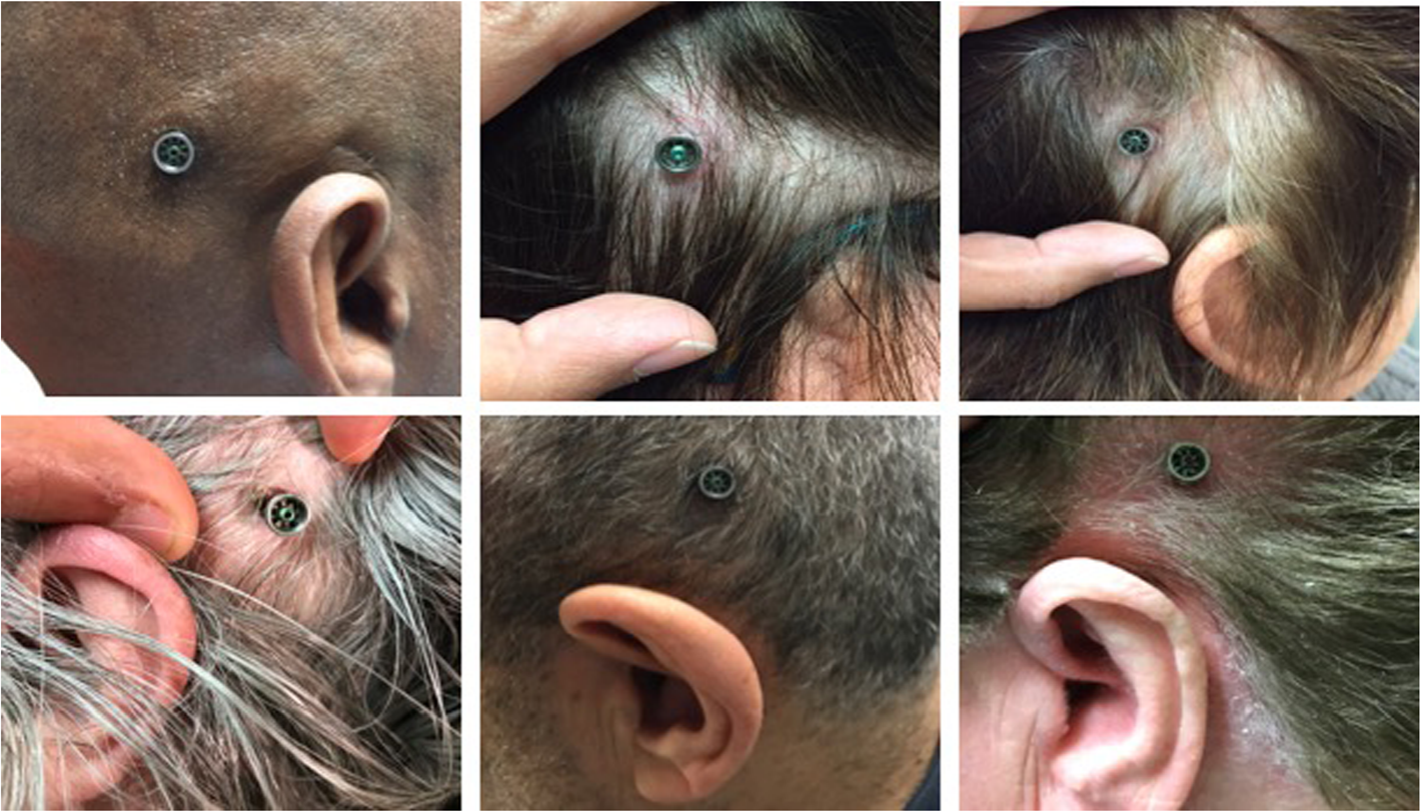
A sample of follow-up abutment site pictures taken at the parallel research clinic most recent visit

The mean (SD) soft tissue status score averages using the IPS Scale were low for inflammation 0.1 (0.1), pain 0.1 (0.1), skin height 0.2 (0.1) and total IPS score 0.4 (0.3) indicating minimal soft tissue changes. There was good inter-rater reliability with these scores. Table 3 shows the summarized IPS results. Digital photographs corroborated these findings. Figure 1 shows a sample of such images.

**Table 3:** A summary of the IPS-scale soft tissue status for patients at their most recent follow-up where they were seen by 3 independent raters

	Inflammation			Pain			Skin Height			Total Score		
Rater	1	2	3	1	2	3	1	2	3	1	2	3
Score	0.3	0.1	0.0	0.2	0.0	0.0	0.3	0.1	0,2	0.8	0.2	0.2
Average	01			0.1			0.2			0.4

There were few minor complications as can be seen by the soft tissue score. The most common intervention in the early post-operative period was the prescription of topical antimicrobial drops on an ‘as required’ basis should the patient experience redness or irritation. Later complications noted included 3 patients with abutment length changes, the requirement of stay sutures inferiorly and skin redundancy superiorly. Most importantly, there were no failures to osseointegrate and no fixtures had been lost in this cohort at the time of last follow-up.

**Fig. 2:**
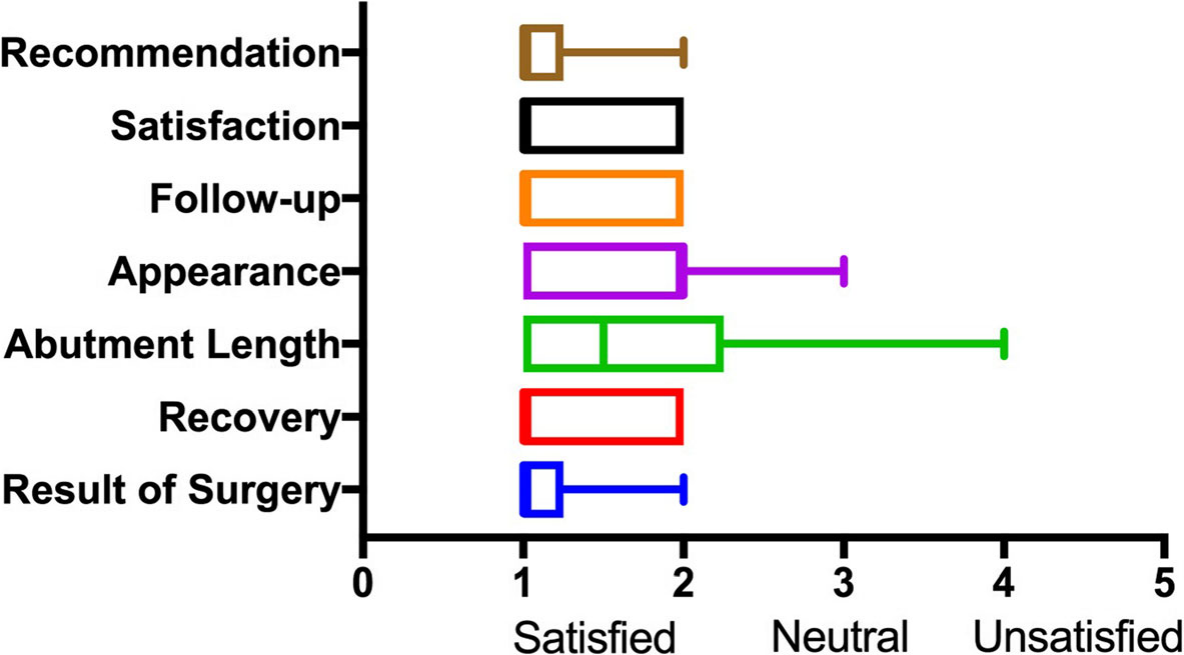
A box and whisker plot showing survey results from the modified surgical questionnaire for MIPS at latest follow-up

In terms of survey data, median (M) and Interquartile Range (IQR) from the MIPS modified SSQ-8 were: result of surgery 1 (1), recovery 1 (1, 2), abutment length satisfaction 1.5 (1, 2), appearance of screw 2 (1, 2), follow-up received 1 (1, 2), overall satisfaction 1 (1, 2), and recommendation of MIPS to others who would benefit 1 (1). Figure 2 presents a graphic summary of modified SSQ-8 survey data.

The patient experiences with MIPS were overwhelmingly positive. All patients reported speedy recoveries, often getting back to routine activities the next day. The surgical experience was felt to be minimized, with patient’s reporting they received high quality care. Issues reported included getting used to having a foreign object on their head and initial maintenance of the implant site. Some patients reported wearing their devices with a degree of selectivity, only using them in more challenging listening environments. A segment from a semi-structured patient interview is included below:

*“I had a screw failure with my previous bone conduction surgery. It was a much better experience with MIPS when I was awake. There was virtually no recovery afterwards – I went shopping 1 hour later. This was compared to my previous surgery with lots of bleeding, bandages and overnight admission. I wear my device all the time.”*

## Discussion

As clinicians, we sense a responsibility to carefully assess and evaluate new developments in patient treatment to ensure that the best quality of care continues to be delivered. The MIPS technique is different from the traditional open approach with which most surgeons are familiar, in a number of ways. Visual access to the bony skull is limited to the narrow tract maintained by the cannula.

A steady hand to stabilize the cannula position and fine tactile feedback are needed to confirm that each pass of the drill is aligned with the original 3 mm test hole. Confirmation that the fixture/abutment assembly has also engaged the countersunk hole once the cannula is removed also demands tactile recognition. The assistance of an experienced second pair of hands cannot be over emphasized. Unlike the traditional open approach, it is impossible to do this procedure alone. The operating surgeon must maintain the cannula in a stable position for the short duration of the case. The assistant is responsible for the swift loading of drill bits, the application of saline irrigation to the operative site and ultimately attaching the fixture/abutment assembly to the handpiece.

The drills are also very different from what went before. It is intuitive that prolonged drilling, failure to irrigate sufficiently with saline and malalignment of drilling trajectory could all result in fixture failure. As the cannula can only hold a small volume of saline irrigant, this should be cold and not from the warming cabinet. Drilling time should also be as short as possible to prevent heating. Constant refilling of the cannula between stages and removal of bone dust also seems intuitive. At the very end of the procedure, excessive leverage should be avoided when uncoupling the connection to handpiece instrument from the secured abutment. This maneuver risks disturbing even the most perfect of placements and is particularly risky given the increased length of abutments needed when soft tissues are not reduced.

While keen to adopt this simplified approach, we were equally keen to discover whether such changes were possible without compromise to patient care. After performing a round dozen cases, a self-imposed moratorium was observed until sufficient reassurance had been obtained to justify an ongoing change in practice.

Our study has demonstrated that it is possible to maintain secure fixture/abutment placement with the MIPS procedure. With a minimum follow-up of 1 year, skin complications were low, consistent with other invetigators [4]. Implants were stable, and it is reassuring to us that no fixtures were lost in this highly-scrutinized cohort. We have previously shown that this change in practice comes with benefits of cost efficiency and better resource allocation [2]. Here we show additional value in proven patient satisfaction with the MIPS procedure in addition to the well documented improvements in quality of life for BAHD users [16, 17].

It is likely that both training and case selection will have some bearing on surgical outcome. Repeated practice of the surgical steps in a training model prior to real-time surgery would seem to be a prudent prerequisite to successful fixture placement even for those who consider themselves to be experienced surgeons with the traditional system. It is humbling to acknowledge the natural learning curve of any new procedure no matter how simple it may seem. The consequences of failure are significant to the patient.

As the MIPS procedure is performed without soft tissue reduction, longer abutments are used with the same 4 mm fixture of purchase on the skull. Simple rotational physics dictates that increasing the distance from the fulcrum reduces the load necessary to exert a displacing force. This principle is explained by Archimedes’ Law of the Lever:

$$ \mathrm{Torque}\ \left(\mathrm{N}.\mathrm{m}\right)=\mathrm{Force}\ \left(\mathrm{N}\right)\ \mathrm{x}\ \mathrm{distance}\ \left(\mathrm{m}\right)\ \mathrm{from}\ \mathrm{fulcrum} $$

The thicker the scalp, the longer is the required abutment. We note that all our cohort received an abutment of 12 mm or 9 mm. No cases required the longer 14 mm abutment which still relies on just 4 mm of skull purchase. As other centers report their results, it will be interesting to see whether the 14 mm abutments are as stable as we have found the 9 mm and 12 mm abutments to be. If this is not the case, some discretion in patient selection may be appropriate.

A limitation of this study relates to the modified SSQ-8 use for MIPS. Although the SSQ-8 has been independently validated for urogynecological surgeries, the adapted form used for this study has not been studied in the context of otologic surgeries. The survey data was congruent with the interview results, with both methods suggesting encouraging experiences with MIPS. This at least provided the reassurances we were seeking from a quality assurance point of view.

The study’s strengths include a long follow-up period for what is still a relatively new technique to North America. With a minimum of 1 year follow-up we anticipated adequate capture of both short and long-term complications. The study also allowed for a robust evaluation of MIPS since objective surgical and subjective patient factors were assessed. The sample size (*N* = 12) for the semi-structured interview was adequate to achieve thematic saturation [18]. The patients will continue to be followed and watched closely to ensure ongoing stability.

## Conclusion

As one of the first North American adopters of the MIPS procedure, we thought it diligent to perform a quality assurance project using our own original prospective cohort. Our findings conclude both device stability and patient satisfaction with no loss of fixtures.

Consequently, we have adopted MIPS as our procedure of choice for the placement of all percutaneous BAHDs.

## Abbreviations

BAHD: Bone Anchored Hearing Devices
BCHI: Bone Conduction Hearing Implants
HCP: Healthcare Providers
IPS: Inflammation Pain Skin height
MIPS: Minimally Invasive Ponto Surgery
OR: Operating Room
SSQ: Surgical Satisfaction Questionnaire
